# Integrating Sensory/Actuation Systems in Agricultural Vehicles

**DOI:** 10.3390/s140304014

**Published:** 2014-02-26

**Authors:** Luis Emmi, Mariano Gonzalez-de-Soto, Gonzalo Pajares, Pablo Gonzalez-de-Santos

**Affiliations:** 1 Centre for Automation and Robotics (UPM-CSIC), Arganda del Rey, Madrid 28500, Spain; E-Mails: mariano.gonzalez@car.upm-csic.es (M.G.-S.); pablo.gonzalez@car.upm-csic.es (P.G.-S.); 2 Department of Software Engineering and Artificial Intelligence, Faculty of Informatics, University Complutense of Madrid, Madrid 28040, Spain; E-Mail: pajares@ucm.es

**Keywords:** agricultural autonomous system, computer-vision, precision agriculture

## Abstract

In recent years, there have been major advances in the development of new and more powerful perception systems for agriculture, such as computer-vision and global positioning systems. Due to these advances, the automation of agricultural tasks has received an important stimulus, especially in the area of selective weed control where high precision is essential for the proper use of resources and the implementation of more efficient treatments. Such autonomous agricultural systems incorporate and integrate perception systems for acquiring information from the environment, decision-making systems for interpreting and analyzing such information, and actuation systems that are responsible for performing the agricultural operations. These systems consist of different sensors, actuators, and computers that work synchronously in a specific architecture for the intended purpose. The main contribution of this paper is the selection, arrangement, integration, and synchronization of these systems to form a whole autonomous vehicle for agricultural applications. This type of vehicle has attracted growing interest, not only for researchers but also for manufacturers and farmers. The experimental results demonstrate the success and performance of the integrated system in guidance and weed control tasks in a maize field, indicating its utility and efficiency. The whole system is sufficiently flexible for use in other agricultural tasks with little effort and is another important contribution in the field of autonomous agricultural vehicles.

## 1. Introduction

Autonomous outdoor navigation of vehicles with integrated sensor and actuation systems was proposed in the 1920s, but it was first realized in the 1980s, when the technology was mature enough to allow for actual tests [1]. Currently, there exists a growing interest in the field with significant progress [2]. NavLab was one of the first and more outstanding vehicles capable of navigating in a real, dynamic environment with the help of machine vision, a range finder and heavy computing power onboard the vehicle [3]. A few years later, some researchers tried to automate agricultural vehicles by using different concepts and techniques. Erbach *et al.* [4] proposed a static system based on radio beacons to triangulate the vehicle's position for steering purposes. A similar system using cameras in the field to track a visual mark on the vehicle was also used to determine the position of the tractor [5]. Although this system was successful, researchers returned to the NavLab philosophy by putting cameras onboard the vehicle. The static vision system evolved toward mobile equipment that was able to identify the environment and use its features for vehicle steering purposes. This technique led several researchers [6,7] to develop controllers for autonomous agricultural tractors to track straight crop rows.

A different approach, based on GPS (Global Positioning System), was proposed by O'Connor *et al.* [8] at around the same time. These authors demonstrated how an autonomous vehicle equipped with a carrier phase GPS with four antennas can provide both position and heading in the field with accuracy high enough to accomplish agricultural tasks. Since then, GPS has been adopted as the typical technique for measuring and controlling a vehicle's position and heading, and it has been included in some commercial systems [9]. Nevertheless, research is still ongoing, and new approaches using GPS for the autonomous guidance of tractors have recently been proposed [10].

Although GPS technology provides good accuracy for guiding agricultural vehicles, machine vision has been shown to be crucial to identify environmental particularities and obstacles; therefore, both techniques started to be merged in the 2000s and became the standard approach for agricultural vehicles [11]. Specifically, camera-based systems have been developed for guidance as the main task [12,13] and for weed and crop discrimination, where guidance was a consequence [14–19]. Guidance and detection tasks require sensors and elements to be conveniently arranged, adjusted, and calibrated onboard the vehicle for accuracy during implementation [20,21].

Apart from the problems of making a vehicle autonomous, an agricultural system needs to be equipped with the proper implements to carry out farming tasks such as tilling, planting seeds, weed control, fertilizing, applying pesticide, mowing, and harvesting. This is a step forward in the automation of agricultural systems, which this article's authors are focused on. There are two basic types of implements depending on whether they are carried or towed by the vehicle. The positioning accuracy of the former type depends on the positioning accuracy of the perception system and the vehicle, and most current developments fall in this topic. The latter depends on the accuracy of the perception system, the positioning accuracy of the vehicle, as well as the positioning accuracy of the implement steering mechanism. Some attempts have been made in positioning this type of system by using two GPS antennas, one located on the vehicle and the other on the implement. For example, Nørremark *et al.* [22] developed an unmanned hoeing system for intra-row weed management based on real-time kinematic (RTK) GPS techniques. The system was composed of an autonomous vehicle and a side-shifting arrangement fixed to a weed implement. Both the vehicle and implement were equipped with RTK-GPS; thus, the two subsystems provided their own position to allow the vehicle to follow predefined GPS paths and the implement to act on every plant, whose positions were obtained automatically during seeding. Other effective actuation systems (cultivator and sprayer) have also been developed with mechanical control based on the RTK-GPS perception system [23].

One important concern in agriculture is productivity, where agricultural tasks have to be carried out with accuracy, maximum performance, and minimal resources. This situation means that the integration of the aforementioned systems (the vehicle and the implement) must be carried out under an architecture with an effective and reliable design to meet all requirements, specifically the expected real-time performance. Thus, the architecture is a crucial issue, where all subsystems are to be coordinated. Suprem *et al.* [24] highlighted the importance of the effective integration of sensors, computers, and actuators. There has been a great emphasis on the development of the individual elements but not so much on their integration; note that integration is particularly crucial in agricultural vehicles where the ideal situation is to design flexible and open systems for more than one agricultural application, as mentioned by [25], with the aim of making full use of agricultural vehicles for a wide range of agricultural applications. Accordingly, García-Pérez *et al.* [26] proposed a hybrid agent-based architecture for agricultural purposes, where perception and actuation tasks are integrated and conveniently coordinated.

Slaughter *et al.* [27] reviewed systems in autonomous robots for agricultural tasks and identified four main subsystems: guidance, weed detection and identification, precision actuation, and mapping. Guidance and weed detection and identification are based on RTK-GPS and imaging sensors. Actuation systems are focused on precise control where weeding is a specific agricultural treatment [28], based on micro-sprays, cutting tools or thermal and electrocution devices. Mapping is the process of applying information obtained at a previous stage to the application; Bak and Jakobsen [29] obtained a map during sowing, which was used during the treatment of weeds. These four subsystems are also described in [2].

On the other hand, Rovira-Mas [30,31] proposed an open architecture for intelligent autonomous vehicles, based on a three-layer (safety, information and machine actuation) structure. The safety layer is responsible for all security aspects concerning the vehicle and the user's integrity. The information layer (perception) is in charge of processing all data supplied by the set of sensors onboard the tractor. Finally, the actuation layer (action and decision making) executes the decisions made according to the intelligent processes. All of these layers are interrelated to fulfill the difficult agricultural requirements. It is clear that, for the progress of agricultural autonomous vehicles, it is necessary to follow an architectural model based on such schemes with the required flexibility and scalability to expand the range of the vehicle's applications while simultaneously achieving adequate robustness and efficiency. Rovira-Mas [30] developed the perception system in depth, emphasizing the sensor deployment under a specific configuration for real-time purposes, as well as analyzing the following four important properties: flexibility, scalability, robustness, and efficiency.

**Figure 1.**
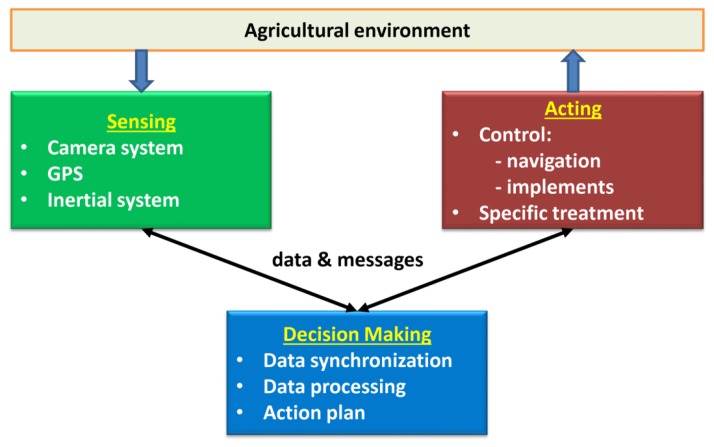
Architectural design: perception (sensing), actuation, and decision making.

Based on the above considerations, we propose an architecture that integrates the above four subsystems (guidance, weed detection and identification, precision actuation, and mapping) while covering the above-mentioned four properties (flexibility, scalability, robustness, and efficiency). This process is achieved with the proposed scheme displayed in Figure 1. It consists of three main modules: sensing, acting, and decision making. Sensing is in charge of collecting information from the environment through the set of sensors available (imaging, inertial systems, and GPS). The information must be appropriate for guidance, weed/crop detection and identification, and mapping. Sensors are adapted according to tasks to be carried out, and new sensors could be added when required, such as range finders for safety navigation (flexibility). Depending on the agricultural application, each sensor can be replaced by similar sensors with different specifications (scalability). The harsh environmental conditions must be determined by sensors (robustness). All sensors must be able to provide data to be synchronized for real-time implementation (efficiency). The Decision-Making System is in charge of processing the information through specific procedures and algorithms for guidance, weed detection and identification, and mapping. The hardware/software components are designed with the aim of receiving all available information supplied by the sensors, which can be activated/deactivated conveniently (flexibility, scalability). These components control all data and processes to guarantee that they are received on time (robustness) with the required coherence for real-time applications (efficiency). After this, decisions are made to be transmitted (messages) to the perception and/or actuation systems when required. Control actions are applied either for navigation or on the agricultural implements for specific tasks, such as weeding. Different implements should be possible, and different parts of the implements can be activated or deactivated (flexibility and scalability). Implements must act with the highest precision as possible for site-specific applications (robustness and efficiency). All subsystems are linked with the appropriate communication protocol. The proposed architectural design with the separated functionalities and properties is the main contribution of this paper. The open architecture allows the same robot to be used for different agricultural applications with little effort, one of the main demands of manufacturers and farmers. We have successfully tested this design in maize fields with a high degree of performance.

**Figure 2.**
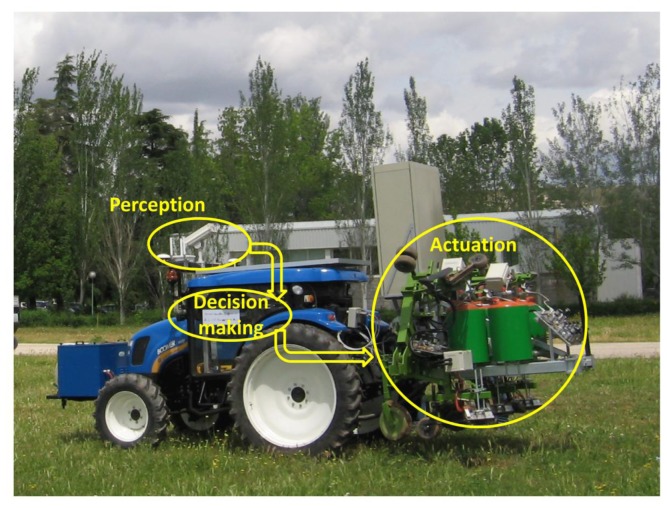
Perception, decision making, and actuation in an agricultural vehicle.

Thus, this paper aims to configure a whole system integrating perception, actuation, and decision making as subsystems for an agricultural autonomous system working on real wide-row crops (see Figure 2). Our proposed architecture is designed for weed control in maize fields [32], but it should be suitable, using the same vehicle with its architecture, for other agricultural purposes such as cereal treatments or even for seeding.

The paper is organized as follows: Section 2 presents the perception system that relies on machine vision. Its main tasks are row identification and weed detection. Section 3 briefly describes the actuation system. Then, the integration of the perception and actuation as well as the decision-making mechanisms are explained in Section 4. Experiments have been conducted in a real maize field with an autonomous vehicle that is a part of the RHEA project (robot fleets for highly effective agriculture and forestry management) in which the subsystems introduced in this article are integrated; Section 5 details those experiments and discusses some results. The conclusions are finally drawn in Section 6.

## 2. Perception System: Localization and Weed Detection

The perception system is designed for crop/weed identification with two main goals: row following and weed discrimination for site-specific treatment. Crop row detection is the basis for both weed discrimination and guidance and requires the localization and identification of the rows (straight line equations) in the image. This section is devoted to the specification of the perception system, the steps for data processing, and the properties of the system architecture.

### 2.1. Description of the Perception System

**Figure 3.**
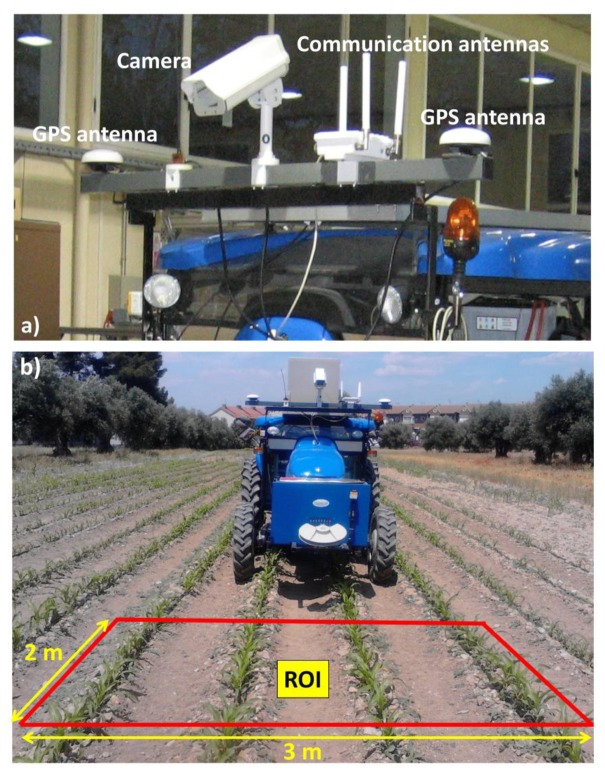
(**a**) Sensors on-board the tractor; (**b**) Region of Interest (ROI) for the vision system.

**Figure 4.**
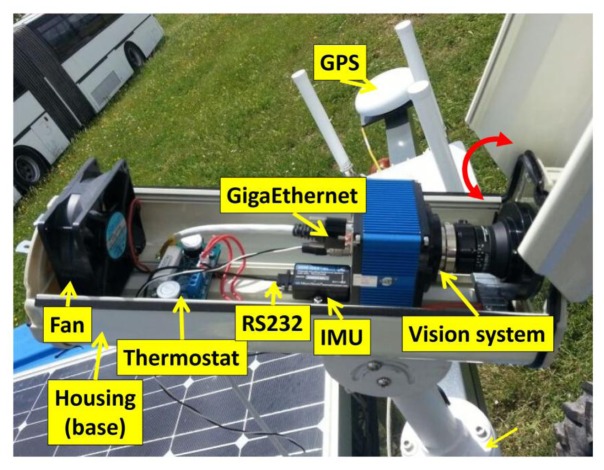
Perception system: sensors and equipment.

The perception system consists of three main sensors: color camera, Inertial Measurement Unit (IMU), and RTK-GPS (consisting of two antennas: one for XYZ positioning and the other for heading calculations). Figure 3a displays the main sensors on-board the vehicle based on a commercial tractor chassis. The camera and IMU are embedded into a housing with a fan controlled by a thermostat for cooling purposes, assuming that some agricultural tasks are conducted under high working temperatures, above 50 °C. The housing is IP65 protected to work in harsh environments (exposure to dust, drops of liquid from sprayers, *etc.*). Additional to the appointed sensors, the perception system also comprises a wireless communication device, to enable the user for remotely controlling and monitoring the entire system. Figure 4 displays the assemblage of these parts.

The camera-based sensor is the SVS4050CFLGEA model from SVS-VISTEK [33] and is built with the CCD Kodak KAI 04050M/C sensor with a GR Bayer color filter; its resolution is 2,336 by 1,752 pixels with a 5.5 by 5.5 μm pixel size. This camera is Gigabit Ethernet compliant connected to the Main Controller (see Section 4). The IMU (see Figure 4), of LORD MicroStrain^®^ Sensing Systems (Williston, VT, USA) is a 3DM-GX3^®^-35 high-performance model miniature Attitude Heading Reference System (AHRS) with GPS [34]. It is connected via RS232 to the Main Controller and provides information about pitch and roll angles. Both the camera and IMU are robust enough and exhibit sufficient capabilities for real-time performance, required for agricultural tasks. The GPS receiver is the Trimble^®^ BX982 GNSS receiver, supporting two antennas for precise heading calculation, mounted on the lateral ends of the vehicle (see Figure 3a). The goal is to apply specific treatments in the Region of Interest (ROI) in front of the tractor, which is a rectangular area 3 m wide and 2 m long (see Figure 3b). It covers four crop rows in the field. This area starts at 3 m with respect to a virtual vertical axis traversing the center of the image plane in the camera, i.e., where the scene is imaged.

**Figure 5.**
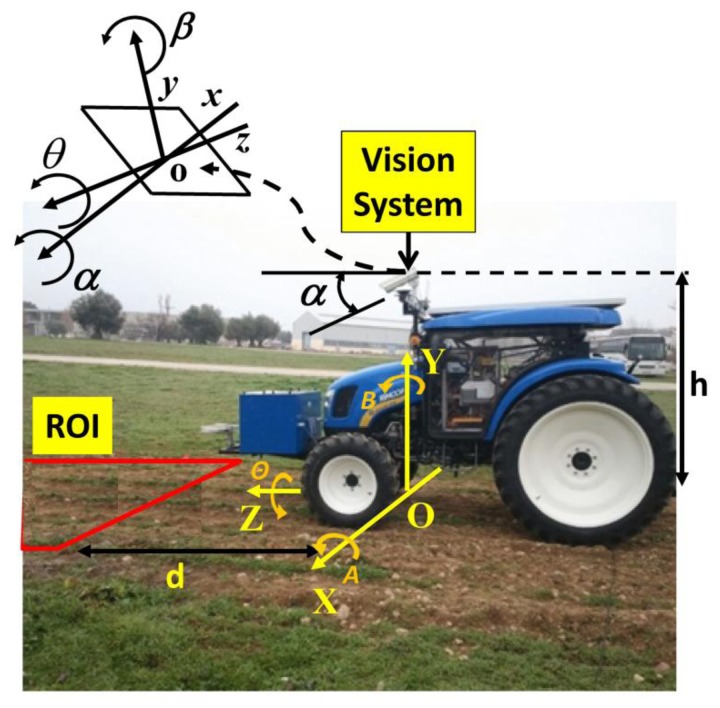
Camera system geometry.

Figure 5, displays the camera system geometry [17]. *OXYZ* is the reference frame located in the ground with its axes oriented as displayed, aligned with the reference axis of the GPS system; h is the height from *O* to the origin *o* of the reference frame *oxyz* attached to the camera; roll (*θ*), pitch (*α*), and yaw (*β*) define the three degrees of freedom of the image plane with respect to the referential system; d is the distance from the beginning of the ROI measured perpendicular to X axis.

### 2.2. Characteristics of the Perception System

Four properties have been identified as basic requirements for the perception system integrated into the proposed architectural design:

(a) Flexibility/Modularity
  The perception system consists of the elements described above with a direct communication link to the Decision-Making System running on the Main Controller. Different sensors can be connected via GigaEthernet and RS232 communication ports, which are standard interfaces. Any sensor can be connected/disconnected without restrictions other than the physical capacity of the Main Controller (see Section 4). The operations of linkage and decoupling can be carried out via software, without affecting the remaining modules or the operability. This procedure allows a multi-sensor arrangement according to the required agricultural tasks. Some crop line detection algorithms do not need the IMU, and when this happens, this sensor is simply ignored and then activated when required. This method avoids important disruptions to the designed systems and at the same time proves the flexibility and modularity of the proposed architecture.
Scalability
  Sensors and their corresponding drivers can be added to increase the amount of work according to the demanded agricultural tasks. Again, the unique restriction is the limitation of the number of ports available in the Main Controller, which can be easily expanded. So, we could add a multispectral or thermal camera for plant discrimination or a stereoscopic vision-based system with a GigaEthernet connection for object detection for safety purposes. Different cameras with higher resolutions are accepted when required. Regarding the processing of data provided by the sensors, the Decision-Making System implemented in the Main Controller allows for high-performance processing.
(c) Robustness
  The camera-based system is robust enough to support the adverse outdoor agricultural environments with sufficient physical and electronic protections. It is designed to withstand mechanical vibrations from the tractor's engine, soil roughness, extreme temperatures, and high variability in illumination. Additionally, the camera is equipped with an UV/IR filter to cut spectral ultraviolet and infrared radiation, which considerably affects the image quality. The IMU is encapsulated and calibrated to provide stable values.
(d) Real-Time/Performance
  The perception system, particularly the camera, is arranged onboard the tractor to cover the region of interest where the specific actuation is to be applied. It is placed closed to the center of gravity to minimize vibrations and undesired mechanical effects. The image acquisition is controlled through the exposure time, again with the aim of achieving high quality images. The data processing for crop/weed detection and guidance is designed under specific modules, programmed as dynamic-link libraries (DLL) in C++, and embedded in the Main Controller. The modules are optimized to work in real-time for the proposed specific treatment.

### 2.3. Process: Integration of Information

**Figure 6.**
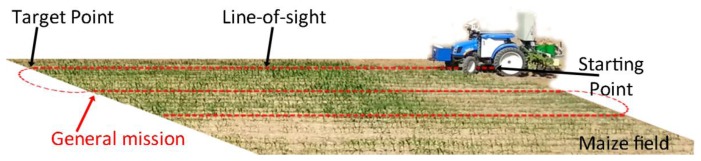
Example of path planning in the maize field.

The vehicle is programmed to follow a pre-defined plan or general mission (see Figure 6). The mission consists of a set of waypoints that are established based on GPS coordinates, which define the beginning (starting points) and end positions (target points) for crossing the field. For this particular case, the information from the mission contains some uncertainty, so that the start and end points may not match the corresponding crop line's center. This requires the use of the crop row detection system to correct this uncertainty presented in the general mission.

In addition to the crop row detection, the perception system delivers information on the weed infestation in the field, allowing the computation of the percentage of weed presence inside the ROI. To do this, the ROI is divided into rectangular sections 0.375 m wide (corresponding to split in two the inter-row space, with the objective to identify the infestation on each side of the crop row) and 0.25 m long (which corresponds to the minimum resolution for the execution of the treatment). The size of these sections is adjusted based on the characteristics of the actuation system that performs the treatment (see Section 3.1). The procedure is as follows:

(1) The operation speed set for this type of treatment is defined as 0.83 m/s (3 km/h).
(2) The pre-defined plan determines the traversal order of the waypoints to be visited, including starting and target points.
(3) Between two waypoints inside the field, the vehicle follows the line-of-sight.
(4) The camera captures images at frame rates up to ten images per second.
(5) The system reads the GPS coordinates at a rate of 10 Hz and captures an image whenever the vehicle moves within 2 m on the field, which is the length of the ROI.
(6) The camera vision system processes each image to identify four crop lines. The IMU provides information about extrinsic camera parameters, pitch (*α*) and roll (*θ*), so that, together with the remaining extrinsic and intrinsic parameters, four expected crop lines are identified. The expected crop lines serve as guidelines to determine the real crop lines [17].
(7) Based on the relative positioning of the two central crop lines identified with respect to the center of the image, if deviations occur between the detected crop lines and the line-of-sight, the lateral deviation and heading are corrected to align the tractor with the real crop lines in the field.
(8) The detected crop lines are used to determine the weed coverage inside the ROI, based on the green densities around the crop lines and between adjacent crop lines [19].

#### Georeferencing Images

Because the raw image received from the camera is transmitted by Ethernet (non-deterministic procedure), the image analysis process consumes a specific amount of time and the actuation point (implement elements that perform the treatment) is located several meters behind the ROI, an element for synchronization and georeferencing is needed to maintain a real-time performance and high-accuracy treatment. To accomplish this synchronization, each image captured is associated with a GPS position, and a map for weed coverage is created that is referenced to GPS coordinates.

## 3. Sensing/Actuating in Crops

Actuation in crops, which is the primary objective in agriculture, is normally performed by implements or devices towed/pulled by tractors that provide energy and motion. Implements are tools composed of simple sensors to obtain the status of the crop and simple actuators to perform simple actuation such as opening/closing valves, moving prismatic cylinders, *etc*. Most of these sensors/actuators are controlled by using PLCs or similar devices. If sophisticated sensor systems such as a machine vision system or range finders are required, they are provided as external devices as indicated in Section 2.

### 3.1. Description of the Actuation System

**Figure 7.**
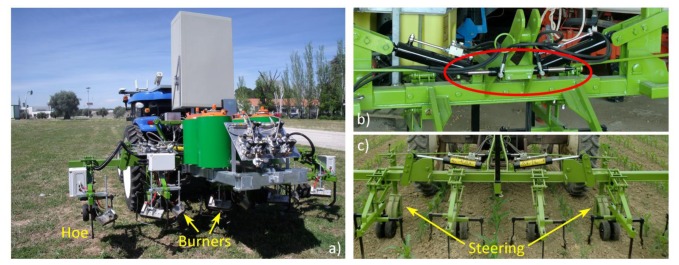
(**a**) Mechanical-thermal machine; (**b**) Implement steering actuator (implement front view); (**c**) Main frame (implement rear view).

Although the proposed system can be applied to a large number of implements, we will specify the system for a particular mechanical-thermal machine [32], which is devoted to weed control (see Figure 7a) in flame-resistant crops such as maize, onion, and garlic. This implement is pulled by the autonomous tractor, and the Main Controller is in charge of decision making and synchronizing the activation of the treatment as well as managing the lateral position of the implement with respect to the vehicle's position.

This specific implement consists of four couples of burners, about 0.25 m long, attached to a main frame (see Figure 7a) to address four successive crop rows. The objective of the burners is to perform selective treatment in the intra-row space. The treatment in the inter-row space is achieved by mechanical actuation, i.e., specialized hoes (see Figure 7a). Every burner's flame intensity depends on the weed coverage identified by the weed detection system presented in Section 2 [35]. The control of the ignition of the burners is performed by the Actuation Controller, which receives the action messages from the Main Controller.

Given that the implement contains two different elements to perform weed control, and given the possible risk of crop damage, an adequate degree of accuracy in positioning the burners and mechanical elements is needed. This step is performed by a guidance system, which is in charge of executing small adjustments in the lateral position of the implement with respect to the vehicle. This lateral positioning system consists of a linear actuator (central double rod hydraulic cylinder, see Figure 7b) that modifies the angle of the steering wheels, allowing the operative machine to move laterally with respect to the vehicle (see Figure 7c). This cylinder features a displacement of ±0.031 m, exerts a force of 2,000 N, and is powered by the hydraulic system of the vehicle. The lateral control of the implement is performed directly by the Main Controller through the controller area network (CAN) bus communication system onboard the autonomous vehicle.

#### 3.1.1. Lateral-Position Sensor

**Figure 8.**
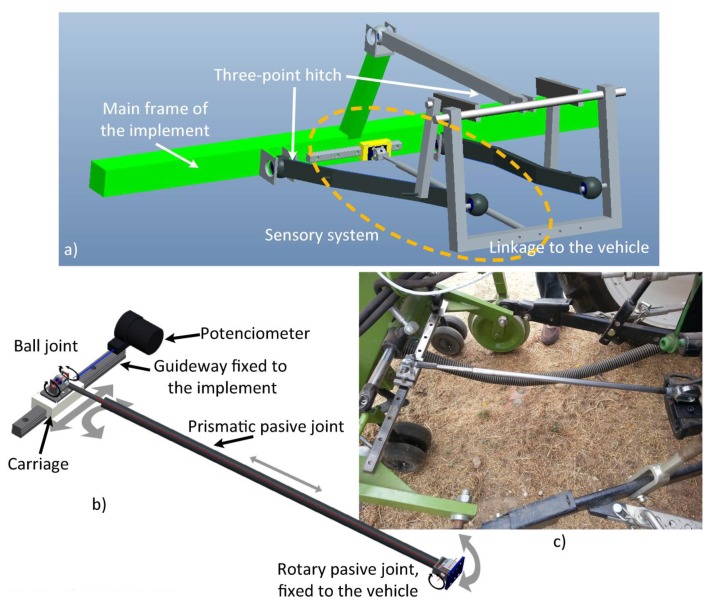
Lateral-position sensor setup. (**a**) Assembly diagram of the sensory system between the implement and the vehicle; (**b**) Structure of the sensory system; (**c**) Photo of actual sensory system.

The positioning device for measuring the lateral displacement of the implement with respect to the vehicle is composed of a passive mechanism and a positioning sensor. The passive mechanism relies on a telescopic arm that joins the vehicle's rear to the implement main frame (see Figure 8b). The end of the arm is fixed to the vehicle through a passive rotary joint, with the rotation axis perpendicular to the arm. The other end is fixed to a carriage through a ball joint. The carriage can slide over a linear guide. A cable-pull potentiometer (JX-PA-20-N14-13S-125) is fixed to the carriage so that the sensor can measure the displacement of the implement. Thus, the positioning device measures the relative displacement of the implement with respect to the vehicle in a direction perpendicular to the vehicle's longitudinal axis. Figure 8b shows the basic scheme of the sensory system, and Figure 8a,c illustrate how it is placed between the implement and the vehicle. In addition to the lateral-position sensor for measuring the displacement of the implement with respect to the vehicle, an encoder (JX-EP-20-N14-110-25C) was placed in the hydraulic cylinder that modifies the angle of the steering wheels for more precise control of the implement guidance.

#### 3.1.2. Measuring the Implement Lateral Position with Respect to the Vehicle

**Figure 9.**
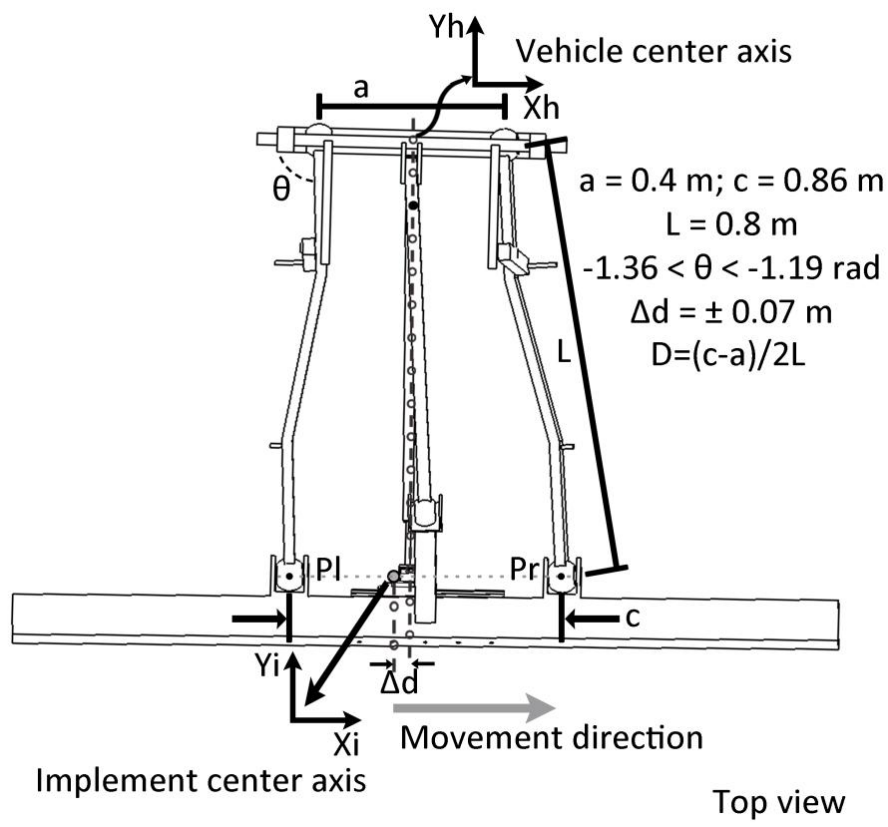
Schematic model of the three-point hitch for connecting the implement.

Based on the three-point hitch geometric model and the sensory system setup, it is possible to relate the potentiometer measurements with the actual lateral displacement of the implement. Figure 9 shows a schematic model of the three-point hitch for connecting the implement, where *Δd* is the difference in the potentiometer measurement and *ΔX* is the real implement displacement with respect to the center of the vehicle. Based on the geometric model of the three-point hitch, Equation (1) and Equation (2) represent the positions of the points *Pl* and *Pr* with respect to the central reference axis *h* (see Figure 9). Equation (3) represents the position of the point *Pr* as a function of *θ* (see Figure 9), which represents the excursion angle of the three-point hitch left arm. Equation (4) represents the implement center axis *i*, as a function of *Pr* and *Pl*. Given the geometric model of the link between the three-point hitch and the implement and knowing the dimensions of the arms and the range of *θ* (see Figure 9), it can be calculated that, in the case of implement maximum excursion, the real displacement *ΔX*, described in Equation (5) is 0.17% greater than the measured displacement *Δd*, described in Equation (7), which is negligible.

(1) $${\left(X_{Pl}-(Xh-a/2)\right)}^{2}+{\left(Y_{Pl}-Yh\right)}^{2}=L^{2}$$

(2) $${\left(X_{Pr}-(Xh+a/2)\right)}^{2}+{\left(Y_{Pr}-Yh\right)}^{2}=L^{2}$$

(3) $$(X_{Pl},Y_{Pl})=\left(\left(Xh-a/2\right)-L\cdot \cos\theta ,Yh-L\cdot \sin\theta \right)$$

(4) (Xi,Yi)=(Pl+Pr)/2

(5) ΔX=Xh−Xi

(6) $$\Delta Y=(Yh-\left(\sqrt{1-D^{2}}\right)\cdot L-Yi$$

(7) $$\Delta \text{d}=\sqrt{{\Delta \text{X}}^{2}+{\Delta \text{Y}}^{2}}$$

### 3.2. Characteristics of the Actuation System

Following the properties described for the perception system in Section 2, the actuation system exhibits the following characteristics:

(a) Flexibility/modularity
  The actuation system consists of a main frame and simple actuators that apply a given process to the crop. In this sense, the main frame and the related positioning systems can be used for a large number of crops, and the specific actuators (burners, nozzles, *etc.*) can be easily changed for different crops. As agricultural vehicles normally provide electric and hydraulic power, there are many different types of actuators that can be used in these devices. This makes the actuation system have little dependence on the type of crop to be treated.
(b) Scalability
  The number of electrical, hydraulic or pneumatic actuators (relays, prismatic cylinders, *etc.*) that can be connected depends on the number of I/O channels provided by the Main Controller. The specific controller used in this work (see Section 4) allows the designers to use a large number of I/O channels that can be even cascade-connected, which makes the number of I/O ports nearly unlimited. Scalability is thus a minor problem.
(c) Robustness
  As the actuation systems consist of a main frame made of steel and a number of commercial actuators that are rugged enough for use in industrial and natural environments, the system exhibits high robustness.
(d) Real-time/Performance
  The lateral positioning of the main frame requires a simple PID controller that does not require high computing power, and signals are activated normally through a CAN bus that has few delays. However, hydraulic valves can have a slow response, jeopardizing the control performance. Thus, real-time performance in actuation systems is critical and must be carefully designed.

### 3.3. Process: Integration of Information

The actuation system consists of two main tasks: (a) controlling the activation/deactivation of the burners and (b) controlling the lateral positioning of the main frame with respect to the vehicle. The first piece of information comes directly from the vision system (camera-based sensor and processing), which informs the exact points where the treatment must be applied based on a weed coverage matrix. The second piece of information comes from the location system (GPS), which indicates the position in which the implement must be a few seconds later, depending on the vehicle's speed, position with respect to the crop lines, and the variations of the heading.

## 4. Perception-Actuation Integration and Decision Making

The perception system is connected and integrated under the Decision-Making System that runs in the Main Controller. Moreover, a part of the actuation system, which is in charge of the implement lateral control, is also integrated under the Decision-Making System. The Decision-Making System is primarily responsible for synchronizing the information coming from the different sensors, to associate the same reference system for each piece of information. Thus, the relationship between what is perceived and where and when the actuation is needed is created.

Other important tasks of the Decision-Making System are as follows: (a) the interpretation of the information; (b) the evaluation of its reliability; (c) the creation of an action plan to be executed by the actuation system; and (d) the supervision of the development of the general mission. Figure 1 displays a schematic of the conceptual architecture followed by the interaction between the perception system, the Decision-Making System, and the actuation system.

### 4.1. Description of the Decision-Making System

The Decision-Making System is in charge of collecting the information provided by the sensors about the environment, producing the actuation plan, and monitoring the execution of the various tasks that make up the general mission. The information must be synchronized, processed, interpreted, and arranged for decision making for the controllers and actuators with the purpose of actuations over agricultural fields. This system is also in charge of sending messages to the sensors and actuators to demand specific behaviors on the corresponding devices. All of these processes run within the Main Controller, which consists of a CompactRIO-9082, with a 1.33 GHz dual-core Intel Core i7 processor, including an LX150 FPGA with a Real-Time Operating System. LabVIEW Real-Time [36] from National Instruments, release 2011, is used as the development environment.

Making Decisions Based on the Reliability of the Information of the Perception System.

Although the commercial devices in the perception system are very trustworthy, some disturbances may occur from the interaction with the environment that can affect the quality of the acquired information. Some of these disturbances include a decrease in the GPS precision or the loss of a message, as well as disturbances in the image due to reflections or lighting changes. Such disturbances can be detected in real time, and based on the degree of influence of the disturbance, an estimation of the reliability of such information can be made.

The failure, loss or alteration of information directly affects the precision with which the treatment is performed at that instant in time, given that in this type of application, the vehicle is in motion and the treatment is being fulfilled based on that movement. For example, in the case of low reliability in the generation of a weed cover map in a small section of the mission, it is better to apply the treatment with the worst-case scenario rather than returning to that specific area.

### 4.2. Characteristics of the Decision-Making System

Based on the above considerations, this paper proposes a design that meets the above requirements under LabVIEW [36], where the four properties identified as appropriate for architectural design in agricultural robots can be summarized as follows:

(a) Flexibility/modularity
  The Decision-Making System receives/sends data from/to the perception and actuation systems when required. They are directly connected to the corresponding devices, and these data are mapped as variables in LabVIEW. All processing and control modules are written as subVIs (functions) that are linked together. The image processing module is a DLL developed in C++ and is also written as a subVI. This means that all modules can be easily replaced, added or removed according to the specific needs of each agricultural application.
(b) Scalability
  The architectural design achieves a high level of scalability because the system can grow simply by adding new functionalities embedded as new modules and connected with internal links.
(c) Robustness
  cRIO supports a high range of working temperatures (0 °C to +55 °C), with ingress protection IP20 and operational humidity up to 90%. This computer supports operational sinusoidal vibrations up to 500 Hz at 5 G. The system has been tested in real, harsh agricultural conditions and can accomplish real tasks with extraordinary robustness. LabVIEW has also been tested successfully in different robotics systems including autonomous agricultural robots. Part of this feature is achieved by suppressing the external links of communication between the different modules in charge of the different devices. Only local protocols are required to arrange ordered data coming from the different systems.
(d) Real-time/Performance
  LabVIEW is specifically designed for running under a real-time operating system. It allows remote communications via Wi-Fi, where limits are established by the Wi-Fi network and not by LabVIEW. This program is suitable for communications with a base station.

### 4.3. Process: Integration of Information

The Decision-Making System is in charge of three main tasks: (a) perception and actuation synchronization; (b) trajectory planning; and (c) weed coverage map interpretation.

The Decision-Making System knows the general mission to be performed, and based on that assignment, the plan that meets the most optimal execution (shortest distance) is fulfilled. This planning consists of sub-paths where the vehicle follows straight or curved lines, linking a starting and a final point. The RTK-GPS is the main sensory system to close the control loop for path following and is corrected when necessary by the camera-based system. The Decision-Making System sends the planned sub-path to the actuation system, which is in charge of the path following.

Along each sub-path, the Decision-Making System commands the perception system to acquire images in coordination with the navigation speed. Each order is triggered every two meters in front of the tractor along each sub-path. This measurement is fixed by the length of the ROI, and the exact positions are provided by the GPS. Full area coverage should be guaranteed to avoid gaps and uncovered areas.

## 5. Results

Various experiments have been conducted to assess the performance of the proposed architecture. The experiments have been carried out in the experimental fields at the CSIC-CAR facilities in Arganda del Rey, Madrid, Spain, on different dates, during May/June/July and October 2013, and additional calibration tests were carried out in December 2013. We have tested our whole system in two different maize fields with sizes of 15 m by 60 m and 18 m by 48 m, i.e., with sufficient lengths to travel along different paths. During the first phase, both the perception and actuation systems were verified separately, and during the second phase, the systems were checked together under the supervision of the Decision-Making System.

### 5.1. Perception System Assessment Tests

As mentioned previously, the operation speed defined for this application was 0.83 m/s (3 km/h) and the ROI was defined to be 3 m wide and 2 m long. These parameters mean that every 2.4 s, the perception system must be able to provide all required data processed and ordered, i.e., with correct synchronization between them. We have analyzed more than 5,000 images with the corresponding GPS and IMU data. A first set of tests was carried out to verify that the data acquisition was synchronized and on time. A second set of tests was intended to verify the accuracy of guidance and weed detection.

#### 5.1.1. Execution Time Tests: Data Acquisition and Processing

Table 1 displays the average time spent in the process of image acquisition and interpretation, beginning with image capture and transmission to the availability of data by the decision-making algorithms. The camera sensor time is split into two parts. The first part is the image acquisition time, which includes the exposure time required to excite the sensor and the time for image transmission to the Main Controller until it is available for the Decision-Making System. The second part is the image processing averaged time, where the computational time differs depending on the density of greenness (crop/weeds) existing in each image.

From the results in Table 1, we can see that the time spent for the perception system (subtotal column) is below the required 2.4 s. Moreover, we have time enough to increase the tractor's speed when required and depending on the specific agricultural task. Minimum frequencies are defined for refreshing data, although normally, the information of the processed image is available when the vehicle travels the operation area segment.

#### 5.1.2. Synchronization Tests: Georeferencing an Image

After confirmation of the ability by the Main Controller to complete the treatment throughout the work area within the time requirements without losing information, the next element to be evaluated is the accuracy of the information obtained by the perception system related to positions on the field, based on the vision system setup in Figure 5. Given the situation presented in Section 2.3.1, the synchronization of one acquired image with respect to a GPS position is not a trivial task and introduces an error associated with the non-deterministic transmission process. The simplest solution is to store the last GPS position available just before sending the command to the camera to acquire an image. This creates an uncertainty regarding whether the acquired image corresponds to the stored GPS position or to a subsequent position, taking into account the age of the stored GPS data, among other factors. A set of tests was carried out following this synchronization approach, where we can draw two interpretations: one related to the error associated with georeferencing the weed coverage matrix (longitudinal error), and another interpretation of the error related to the deviation between the detected crop lines and the line-of-sight supplied by the perception system (lateral error).

**Figure 10.**
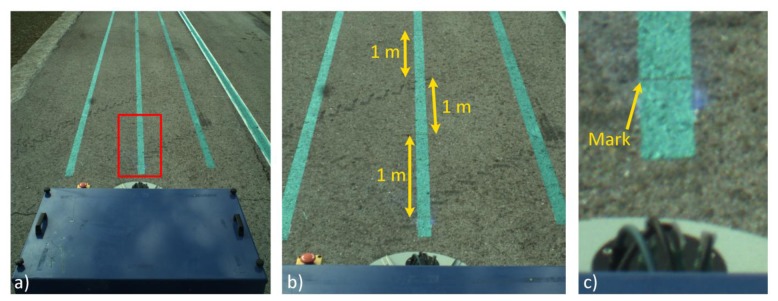
The test conducted for computing the longitudinal error of the vision system. Image acquired and diverse zooms. (**a**) Original image; (**b**) 200% zoom; 1 m apart between marks; (**c**) 800% zoom; mark location: pixel(y) = 1,103.

**Figure 11.**
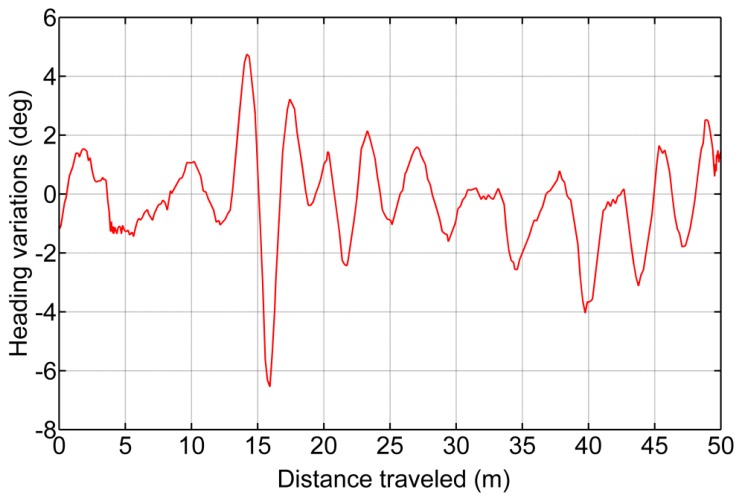
Example of heading variations with respect to the theoretical value when the vehicle is crossing the maize field.

(a) Computing the Longitudinal Error: Weed Coverage Matrix Georeferencing
  Given the vision system setup described in Figure 5, the image acquired was calibrated to associate a point (pixel) in the image as a displacement (in meters) with respect to the reference frame *OXYZ* presented in Section 2.1, shown in Equation (8):
  (8) $$l(y)=4.539E^{-6}\cdot y^{2}-0.0136\cdot y+6.822$$
  This equation is only valid for pixels with the *y* coordinate aligned at the center of the image and within ±1 m from the start of the ROI presented in Figure 5. This calibration has an associated error of ±0.02 m, due to human inaccuracy when selecting the correct pixel and vehicle vibrations transmitted to the frame onto which the camera is fixed.
  Using the vision system characterization, a set of tests was conducted where a series of marks were drawn in a plane soil (see Figure 10) 1 m apart from each other and the vehicle followed a straight line over the marks (approximately 12 m at 0.83 m/s) and acquired images at a rate of 10 fps. Subsequently, the images where the marks were within the valid area were selected and compared with the real location of each mark. The mean square error between the theoretical position of each mark and the estimated position was 0.08 m. This result coincides with the distance between two consecutive GPS positions (at working speed). Taking into account that no synchronization element was implemented except for the matching of the frequency for the GPS and image acquisition, this experimental result validates the assumption that the acquired image must be within ±0.08 m of its associated GPS position in the longitudinal axis.
(b) Computing the Lateral Error: Row Crop Line Detection
  Regarding the error associated with the measure of the lateral displacement of the line-of-sight related to the crop lines detected by the perception system, this error is directly related to changes in the heading of the vehicle in the instant in time when the image is acquired. This heading variation is due to the process of image georeferencing, which entails a translation of the GPS position acquired in that instant in time (corresponding to the position of the CCD sensor, see Figure 5) to the beginning of the ROI. To estimate the associated error, a set of tests was conducted where the vehicle crossed the maize field several times, and using the information generated by the perception system, small adjustments for row following were executed (which generated changes in the heading). Figure 11 illustrates one of the recording changes in the heading of the vehicle, and Table 2 shows the results of all sets. Equation (9) defines the variations in the lateral deviation of the line-of-site based on the variations of the heading:
  (9) Δd=sin(Δφ)⋅L
  where *L* is the distance between the beginning of the ROI and the main coordinate system of the vehicle (which corresponds to the rear axle) and has an associated error of ±0.08 m from the georeferencing procedure.

#### 5.1.3. Image Processing Tests

**Figure 12.**
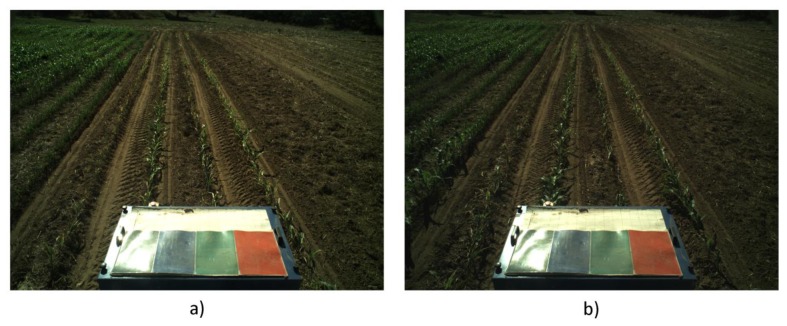
Consecutive original images acquired by the perception system. (**a**) Deviations of the tractor with respect to the crop lines; (**b**) Correction of this deviation.

**Figure 13.**
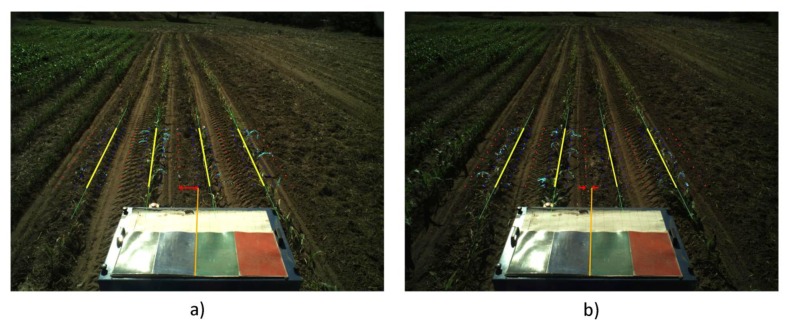
Processed images with the detected crop lines corresponding to images in (**a**) and (**b**) in Figure 12 respectively.

**Figure 14.**
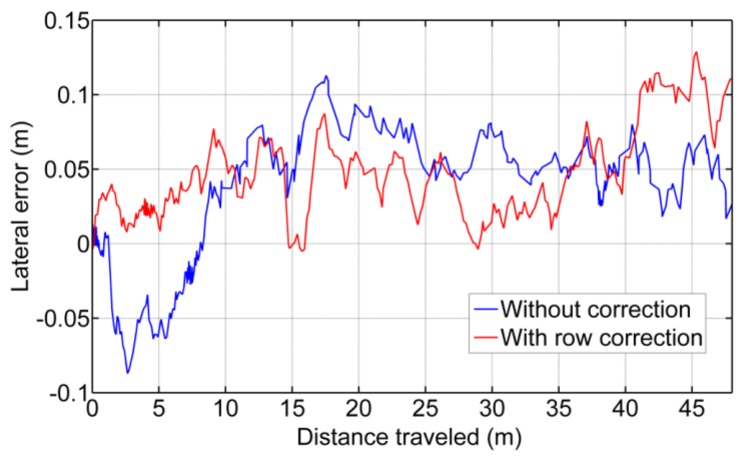
Comparison of the vehicle guidance in a maize field, represented as the lateral error of the rear axle with respect to the theoretical center of the rows.

**Figure 15.**
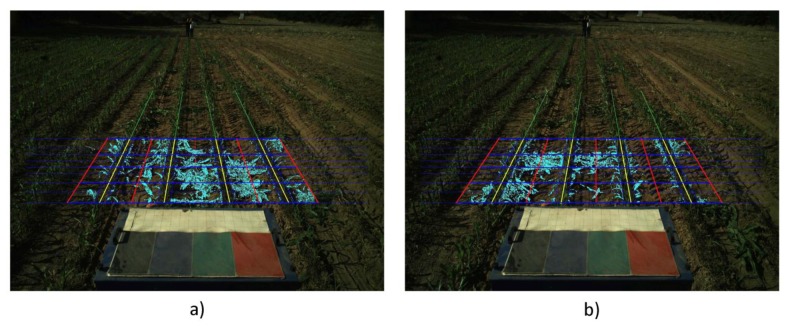
Consecutive images along a sub-path with the detected crop lines (yellow); parallel lines to the left and right crop lines (red); horizontal lines covering 0.25 m in the field.

(a) Correcting the Vehicle Trajectory with Row Detection
  Crop line detection is a crucial task for guidance and weed detection. The first test consists of the analysis of the correct line detection and the tractor's trajectory correction when required. We have randomly selected 400 images acquired during the May/June and October 2013 tests at the CSIC-CAR facilities in Arganda del Rey (Madrid, Spain). The images were acquired over several days under different illumination conditions, i.e., cloudy, sunny days, and days with high light variability. Each processed image is associated with the GPS and IMU data as well as the corrected value for guidance. Given the crop lines, we chose the two central crop lines and determined the correction by computing the deviation of the central line with respect to an imaginary vertical line that divides the image into two equal halves. This deviation was computed in pixels and transformed to a distance based on image calibration [17].
  Figure 12a,b display a sequence of two images acquired during the execution of a straight trajectory following a planned path. Figure 13a,b display the corresponding processed images with the crop lines detected in the ROI. As previously mentioned, the crop lines also serve as local corrections when path planning deviations occur. The tractor in Figure 13a undergoes a slight deviation from the planned trajectory. Indeed, we can see that the upper right corner in the box, belonging to the tractor, is very close to the rightmost crop row and that this box is misaligned with respect to the four crop lines detected in the image displayed in Figure 13a. This misalignment is corrected and can be observed in the subsequent image (see Figure 13b), where the box is better centered relative to the crop lines. This correction is carried out without delays as expected under the proposed architecture. The situation displayed in the above images was normal in our experiments because the tractor navigates on rough agricultural fields with some irregularities.
  From the set of the 400 selected images, we have verified the corrections ordered by the vision system, assuming that corrections below 0.03 m are ignored and that the path following continues with the GPS. After each correction, we verified that the tractor in the next image in the sequence is conveniently positioned. We have verified that on average, a correction has been demanded for 30% of the images (120 images). From these, we have verified that the tractor was correctly positioned on 89% of the subsequent images. For the remaining images, the correction was erroneously demanded. In this case, the path following based on GPS assumed full responsibility of the guidance.
  In Figure 14, the comparison between the use of the information provided by the row detection system and using only GPS for crossing the maize field is illustrated, where it is noteworthy that the row detection system slightly improves the row following, taking into account that the theoretical path to be followed using only the GPS system corresponds to the center of the row by which the two results are compared. It is worth noting that the crop rows at the end of the experimental field were slightly damaged (the last 10 m), due to the large number of tests performed, and in this area, the vision system for row detection produced a large number of errors.
(b) Weed Detection
  For each image, we also compute and store a density matrix of weeds associated with the image. This matrix contains low, medium, and high density values. It is assumed the camera is calibrated and arranged conveniently considering intrinsic and extrinsic parameters [17]. Figure 15 illustrates two consecutive images along a sub-path. They contain three types of lines defining the cells required for computing the density matrix as follows:
  (1) Once the crop lines are identified, they are confined to the ROI in the image (yellow lines), which covers fixed positions in the image.
  (2) To the left and right of each crop line, parallel lines are drawn (red). They divide the inter-crop space into two parts.
  (3) Horizontal lines (in blue) are spaced conveniently in pixels so that each line corresponds to a distance of 0.25 m from the base line of the spatial ROI in the scene.
  (4) The above lines define 8 × 8 trapezoidal cells, each trapezoid with its corresponding area *Aij* in pixels. For each cell, we compute the number of pixels identified as green pixels, *Gij*, (drawn as cyan pixels in the image). We exclude the pixels close to the crop lines with a margin of tolerance, which represents 10% of the width of the cell along horizontal displacements. This is because this margin contains mainly crop plants but not weeds. The weed coverage for each cell is finally computed as *d_ij_* = *A_ij_*/*G_ij_*. The different *d_ij_* values compose the elements of the density matrix.
  From a set of 200 images, we have classified the coverage with three levels (Low, *dij* ≤ 33%, Medium, 33% < *dij* ≤ 66%, and High, *dij* > 66%). These percentages are checked against the criterion of an expert, who determines the correct classification. We have obtained a 91% success rate.

## 5.2. Sensing/Actuation System Test

To measure and validate the accuracy of the sensory system, several tests were performed with the following results:

### 5.2.1. Computing the Error Associated with the Sensory System in Measuring the Lateral Displacement of the Implement with Respect to the Vehicle

**Figure 16.**
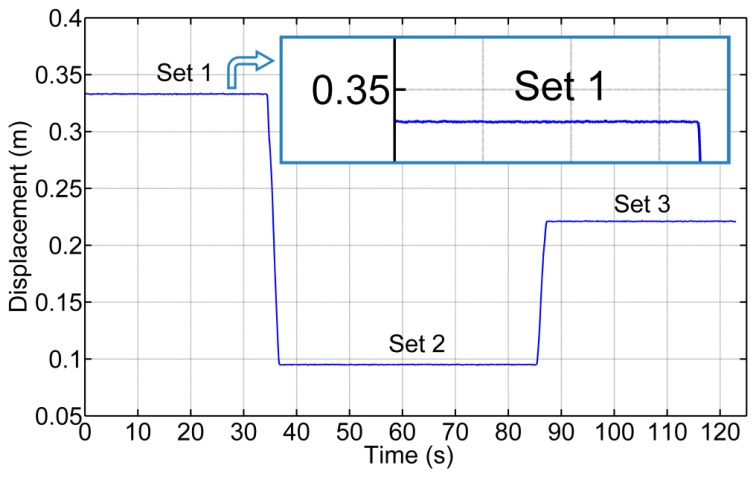
Measuring the position of the sensor in static tests.

**Figure 17.**
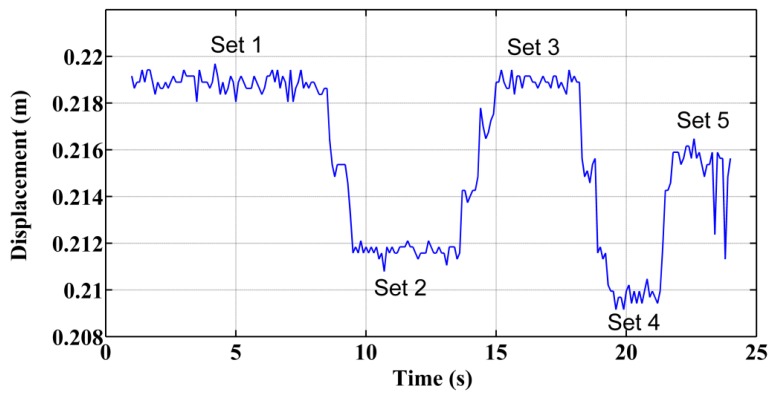
Measuring the position of the sensor where external forces are flexing the sensorial system.

**Figure 18.**
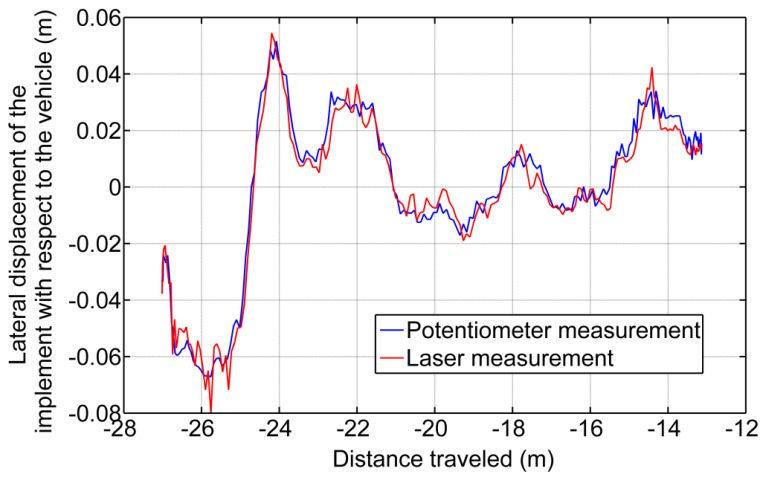
Comparison of the readings of the Laser compared with the output of the potentiometer.

(a) Static Tests: Accuracy of the Acquisition Module
  This trial consisted of acquiring static position measurements from the potentiometer when the implement was placed and anchored at three different displacements with respect to the vehicle. Figure 16 illustrates these three positions as three sets of data. In Set 1 the implement was placed almost in the maximum displacement to the right; in Set 2 the implement was placed almost in the maximum displacement to the left; and, in Set 3 the implement was placed almost aligned with the longitudinal axis of the tractor. This test confirms the proper operation of both the potentiometer and carriage displacement systems and states that the potentiometer measurements have a mean absolute error of ±0.0003 m, given by the standard deviation of the diverse sets (see Table 3).
(b) Deflection Test: Measuring the Error Added by the Rod Bending and the Junctions
  Because the sensory system has a 1 m long arm, the deflection of the arm generates a measurement error. Additionally, the link between the arm and the two joints in both ends has a certain degree of backlash. To measure the error added by this deflection/backlash, the implement was fixed relative to the vehicle and an external force was added to the carriage, generating deflection of the materials and measuring the maximum ranges. Figure 17 shows diverse sets of forces applied to the carriage: Set 1 and Set 3 forces the system to the right, and Set 2 and Set 4 force it to the left. This test does not seek to develop a model of the deflection of the materials that make up the sensory system but instead aims to determine, in the worst case, how much such deflections/backlash affects the final lateral displacement measurement of the implement with respect to the vehicle. Moreover, maintaining a certain degree of deflection in the sensory system reduces the number of system blocks and breaks. The total error due to arm deflection and backlash of the joints is ±0.004 m. (See Table 4).
(c) Dynamic Tests: Measuring the Error of the Entire Sensory System While the Vehicle is in Motion
  A final test was conducted to measure the variations in displacement while the implement is modifying its lateral position with respect to the vehicle and the vehicle is following a straight line at 0.83 m/s. This test defines how much the sensor dynamics affect the control of the lateral displacement. To validate the entire system, a Sick Laser LMS100 pointing to a 0.03 m wide bar located at the center of the implement was installed at the bottom of the vehicle. The laser detects the bar as a peak in the readings, and by measuring the movement of that peak in the transverse axis, it was possible to determine with very high accuracy the position of the implement with respect to the center of the vehicle. Both the laser information and the potentiometer information were related and synchronized with the GPS. Given that the laser was configured to have an opening angle between beams of 0.25 degrees and the bar was 1.2 m apart from the Laser, this validation system had a resolution of 0.0052 m. Figure 18 illustrate the results where the laser measurements are compared with the potentiometer measurements. The mean absolute error calculated was ±0.004 m.

### 5.2.2. Results of the Control for the Adjustment of the Implement Lateral Displacement with Respect to the Vehicle

**Figure 19.**
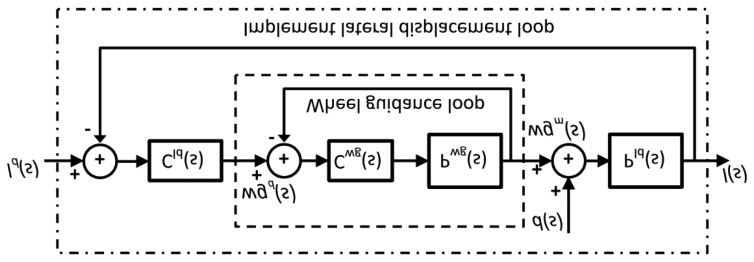
Schematic diagram of the PID controller in cascade configuration for controlling the lateral displacement of the implement with respect to the vehicle.

**Figure 20.**
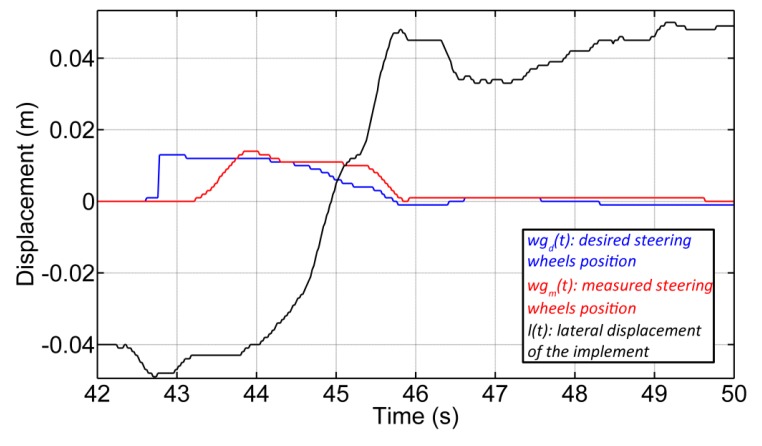
Time response of the two PID controllers.

For lateral control of the implement, two PID controllers in cascade configuration were implemented (see Figure 19). The first controller (*C_wg_*(*s*)) is responsible for adjusting the hydraulic piston that defines the position of the steering wheels of the implement. The desired position of the steering wheels is given by *wg_d_*(*s*), and the current position of the steering wheels, which is measured by the encoder (see Section 3.1.1), is given by the variable *wg_m_*(*s*). The second controller (*C_ld_*(*s*)), given a desired setpoint of the lateral displacement of the implement (*l_d_*(*s*)), generates the desired steering signal. Figure 20 presents the response of both controllers, where the vehicle was moving in a straight line at 0.28 m/s. The response time of the wheel guidance controller was set based on the system that it replaced (an operator with a steering wheel), and the response time of the implement lateral controller depended on the speed of the vehicle.

**Figure 21.**
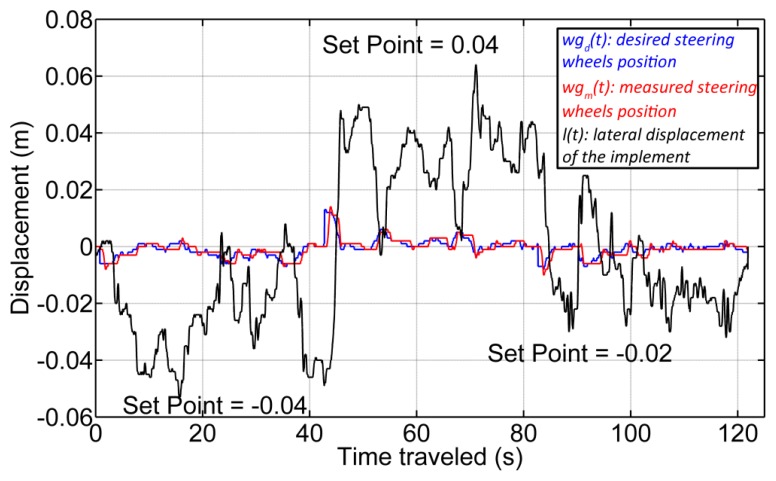
Experimental results of the implement lateral displacement control with three different setpoints.

Figure 21 illustrates an example of the implement lateral-position control with three diverse setpoints. Although it is observed that the implement is not maintaining the desired position for each setpoint due to disturbances from the soil (*d*(*s*)), the results obtained are encouraging. We have been able to keep the implement around the desired position with a mean absolute error of 0.01 m for a complex and heavy system with a very important dynamic response.

### 5.2.3. Calculation of Delays in the Thermal Treatment

As a final test of each individual element presented in this article, the calculation of delays associated with the treatment for weed control is presented. A set of tests was performed to detect in which GPS position the burners turn on and off, using an Internet protocol camera (IP camera), model Axis 211M, mounted on the back of the implement pointed to a couple of burners (set at a rate of 10 fps). The method to associate an image with a GPS position is the same synchronization approach presented in Section 5.1, which has an error of ±0.1 s. For conducting such tests, a straight path was defined, and within that path, a set of points where the burners were turned on and off consecutively was selected (see Table 5). The same criterion used by [35] was applied to determine in which image the burner could be considered ignited or extinct.

**Figure 22.**
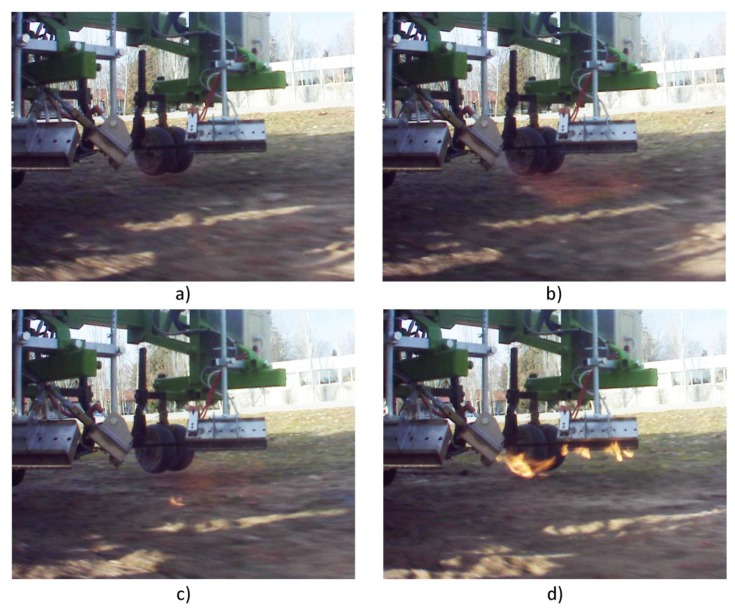
Example of the image sequence used for the calculation of the delay of the thermal treatment. (**a**) Detection of the ignition starting; (**b**) Detection of flame on the ground; (**c**) Detection of the withdrawal of the flame; (**d**) Detection of the total extinction of the burner.

Figure 22a represents the time right when the burners start to turn on. After three consecutive frames (approximately 0.3 s), the burners are operating 100%, corresponding to Figure 22b, where a uniform combustion throughout the burners can be observed. Regarding the extinction of the burners, Figure 22c represents the time right when the burners start to turn off (where the uniform combustion begins to fail). And, after five consecutive frames (approximately 0.5 s), there is no combustible pressure in the system, and the last remnants are consumed, representing the situation of Figure 22d.

Based on the observation of the acquired images, it was possible to calculate the associated delays of the treatment activation. Each selected image had an associated GPS position, and the position where the command was sent (desired position) was compared to the position where the action for ignition/extinction was effective (real position) for calculating the associated delay (see Table 5). The treatment is considered to begin when the flame makes contact with the soil (see Figure 22b). On the other hand, the treatment is considered to end when the flame stops making contact with the soil (see Figure 22c). In addition, a consumption of fuel in the transition stages presented in Figure 22 can be observed, so these transitions are taken into account when carrying out the study of delays in the system and the comparison between the activation of the actuation system and savings in the product used for the treatment.

With the calculation of the associated delays for both the ignition (0.7 s) and the extinction (1 s) of the burners, the Decision-Making System can adjust the coordinates where such commands must be sent given an operational speed to increase the effectiveness of the treatment.

## 5.3. Perception/Actuation and Decision Making

With the aim to present a complete working system, a general test that involved the coordination between the perception system and the actuation was executed. Given that the system was designed for selective weed control, this final test was used to assess the ability of the system in performing this task.

**Figure 23.**
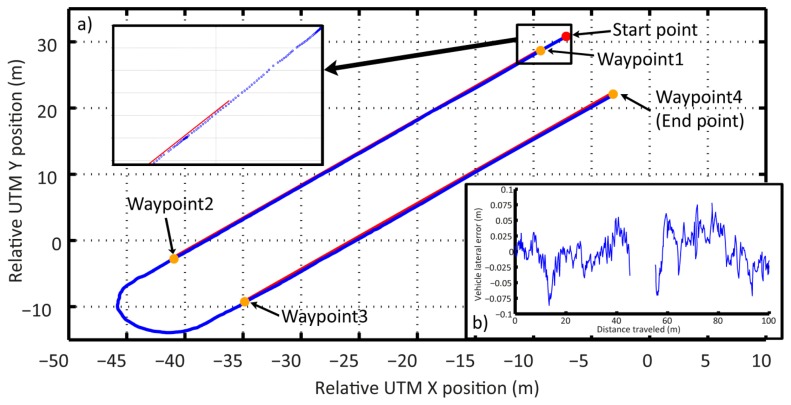
(**a**) The path of the vehicle recorded following the pre-defined mission; (**b**) Error between the trajectory followed by the vehicle and an estimation of the position of the center line of the central rows for each sector.

The whole agricultural system was scheduled to follow a pre-defined plan along the maize field, consisting of the following four waypoints (see Figure 23), which was part of the general mission to be accomplished. The other part of the mission is the execution of the weed control treatment by weed coverage detection, synchronization, and actuation. The primary objective of the mission was to follow the waypoints without putting the crop at risk and to execute an effective treatment in the areas where the weed coverage was higher than the minimum permitted coverage. The Decision-Making System was in charge of this primary objective, based on the information provided by the perception system with the actuation system.

### 5.3.1. Path Following Test: Deviation Errors

The pre-defined path (the four waypoints—red lines) represents the start and end of each pass through the field. The path was selected with a certain degree of uncertainty, i.e., did not correspond exactly to a centered and parallel line between the two central rows. This represents the mapping proposed in [2,27]. Therefore, the perception system must identify the misalignment of the followed path (line-of-sight) with respect to the row crops. Table 6 presents each of the relative UTM coordinates where the row detection system generated an output (every 2 m of distance traveled) and the correction parameter over the line-of-sight followed. Sector 1 corresponds to the straight line that connects waypoint 1 with waypoint 2; Sector 2 corresponds to the straight line that connects waypoint 3 with waypoint 4. Figure 23b illustrates the difference between the trajectory of the vehicle and an estimated position of the center line of the central rows for both Sector 1 and Sector 2.

In this test, the corrections were not made based on the same magnitude as indicated by the row detection system but were decreased by a factor related to the phenological stage of the maize plants, given that the width of the plants could affect the precise detection of the rows (for this test, the phenological stage of the maize was between 5 and 7 leaves with collar). This factor was adjusted to decrease the abrupt changes in the heading of the vehicle given the corrections that must be performed; however, the time and distance needed to converge in following the row crops were increased.

### 5.3.2. Weed Control Test: Amount of Product Applied in the Treatment

The second part of the mission presented in Figure 23 was the execution of the weed control in the maize field by a mechanical-thermal treatment. To do this, each time an image was acquired by the perception system, a weed density matrix was given. This matrix is interpreted by the Decision-Making System to plan the ignition and extinction of the burners. Each matrix cell contains the weed density information (*d_ij_*) of either the left side or the right side of each crop row, in an area of 0.375 m wide by 0.25 m long. Given that the thermal treatment is performed in the intra-row space, the action is executed by each couple of burners. Therefore, the matrix must be processed to define the state of each couple of burners at each instant in time.

**Figure 24.**
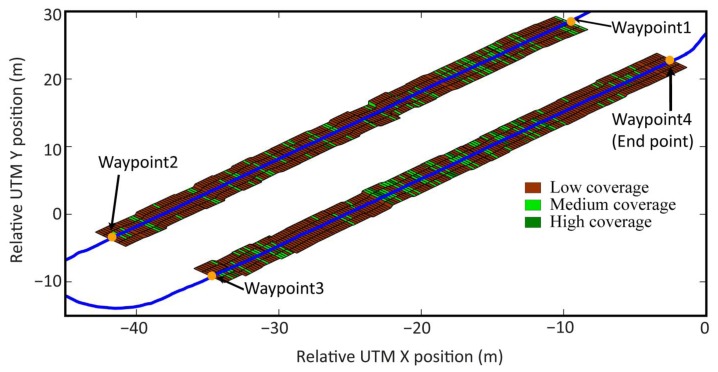
Resulting weed coverage map based on the weed density matrices and the interpretation of the three density levels.

**Figure 25.**
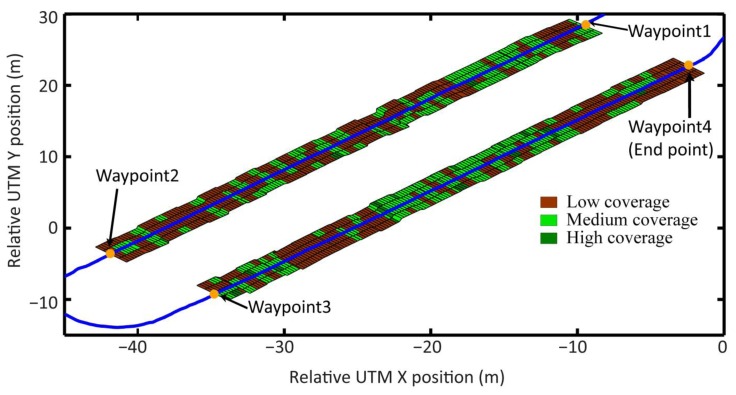
Resulting map of the burner's actuation based on the original weed coverage matrices and the delays associated with the ignition and extinction of the burners.

Figure 24 illustrates the interpretation of the weed matrices and the resultant weed coverage map for the entire mission, when all coverage matrices are joined consecutively. It can be seen that in many cases, the treatment is only necessary in one cell, i.e., in the intra-row space with an area of 0.25 m wide by 0.25 m long, which represents precisely the minimum area defined for treatment. Given the georeferencing errors, the communication delays, and the electro-mechanical limitations of the entire system (limitations of the perception system, the Main Controller, and the actuation system), it is impossible to accurately fulfill the ignition and extinction of the treatment for the worst cases (only one cell). Therefore, to execute an effective treatment, the burners must be turned on early enough to ensure that the desired area is being treated properly. Given the delays calculated in the previous test, the original weed coverage map becomes the map presented in Figure 25.

These electro-mechanical and communication limitations lead to the increase in the use of product for weed control (in this case, LPG). Table 7 presents a comparison of the use of product between a hypothetical treatment (Situation 1) following this mission but without the use of the perception system for weed detection (assuming full weed coverage in the entire mission) and the real treatment (Situation 3) executed with the resulting weed coverage map presented in Figure 25. Table 7 also presents the use of product in a hypothetical treatment (Situation 2) where there are no electro-mechanical and communication limitations.

## 4. Conclusions

The configurations of the perception, the decision-making, and the actuation system have been defined to make up an autonomous vehicle for agricultural applications and, more specifically, for weed control in wide-row crops.

The described perception system has two important tasks: (a) the detection of the crop rows for guiding the vehicle in the field and (b) the detection of weed coverage to perform selective weed control. Based on the tests conducted, the perception system has been shown to be flexible, scalable, robust, and with a suitable level of real-time performance for the application for which it was designed.

Commercial devices were used with standard communication protocols for exchanging messages and interaction between them; the system has proven its reliability for a large number of working hours; the processing algorithms have proven to be quite flexible and scalable, with the ability to adapt to different situations for both the system itself and the environment perceived.

The image acquisition and data processing capabilities have been presented, as well as the required times associated with each task performance and the limitations of each system. The errors associated with the integration of information between image acquisition and GPS positions have been characterized, where positioning the weed coverage matrix has an associated error of ±0.08 m, and the lateral displacement of the path followed with respect to the center of the crop rows has an associated error of ±0.03 m. Both errors are perfectly manageable because the actuation system, i.e., the burners, has a working length of 0.25 m, allowing for a certain degree of flexibility in planning, and allows for minor adjustments on where the burners should be turned on and off. Possible future work to improve the georeferencing of images is the use of an external trigger to synchronize the capture of images, a capability already incorporated in the GPS, the Main Controller, and the camera.

Crop row detection is a crucial task for guidance and weed detection. The proposed method achieves acceptable results with 89% of successful corrections in path following. Additionally, the high weed detection rate (91%) verifies the real-time performance of the proposed approach. This has been achieved under different illumination conditions and different crop growth stages, verifying the robustness and efficiency of the whole system.

A lateral-position sensor device has been designed and integrated into the mechanical-thermal machine to execute the control of the lateral displacement of the implement with respect to the vehicle. The device has proven to be robust enough to work in difficult working conditions (given the amount of dust and water) and with sufficient reliability and accuracy for accurate control. Moreover, the controller designed for executing this task, although it meets the desired characteristics, requires a major adjustment for better rejection of disturbances coming from the contact of the implement with the ground.

The delays associated with the activation of the treatment were computed, i.e., the ignition and extinction of the burners. Given the architecture of the Decision-Making System and the Actuation Controller, in addition to the electro-mechanical delay of the relays and igniters, both the ignition time delay (0.7 s) and the extinction time delay (1 s) were measured. To ensure an effective treatment, the planning of the activations of the burners must take into account these delays, which leads to a higher amount of applied product. However, the tests conducted have shown that selective treatment remains, and depending on the coverage of the weeds in the field and the proximity between patches of weeds, there may be higher or lower product savings. Future work can focus on improvements to reduce the delays associated with communication between the Decision-Making System and the actuation computer. These two elements can be integrated into the same computer, a capability that can be fulfilled by the current Main Controller.

The integration of the sensors and actuators presented in this article has been positively assessed by the RHEA consortium. The experimental results obtained with individual vehicles make the incorporation of this design into a fleet of robots promising, which is the main future objective of the authors.

The proposed architecture, with three main systems, is designed for weed control in maize fields, but thanks to its flexible and open design, the same vehicle can be used for different agricultural tasks. The unique requirement requires the adaptation of new elements and related processes for the intended task. For example, these systems could be used for site-specific treatments in garlic or other crops with different row widths.

## Figures and Tables

**Table 1.** Averaged times required by the perception system until data are available for control and actuation.

	**Camera Sensor**			**GPS**	**IMU**	**Synchronization (Next Treatment Segment)**

	**Image Acquisition**	**Image Processing**	**Perception (Sub-Total)**	**Acquisition**	**Acquisition**	
**Average Time (ms)**	50	250–300	300–350	1	1	2.4 s
**Refresh Rate (Hz)**	10	3.3	2	10	50	-

**Table 2.** Standard deviations of the variations of the heading and the error associated with the calculation of the lateral displacement of the line-of-sight related to the crop lines detected by the perception system.

	**Standard Deviation of the Variation of the Heading *Δφ* (deg)**	**Error associated with the Measurement of the Lateral Deviation *Δd*, (m)**
**Set 1**	0.36	0.03
**Set 2**	0.26	0.03
**Set 3**	0.24	0.02
**Set 4**	0.32	0.03
**Set 5**	0,18	0.012
**Total**	0.27	0.03

**Table 3.** Standard deviation and variance of the data presented in Figure 16.

	**Standard Deviation (m)**	**Variance (m^2^)**
**Set 1**	0.000256897	6.59961E-08
**Set 2**	0.000224263	5.02939E-08
**Set 3**	0.00025533	6.51932E-08

**Table 4.** Mean displacement values of the sensory system due to material flexing.

	**Mean Value (m)**
**Set 1**	0.2189 ± 0.0003
**Set 2**	0.2117 ± 0.0003
**Set 3**	0.2189 ± 0.0003
**Set 4**	0.2087 ± 0.0003

**Table 5.** Relative UTM positions where the ignition and extinction commands were sent, the associated relative UTM positions where the action was detected, and the produced delays.

		**Desired Position (Relative)**		**Real Position (Relative)**		**Time Delay (s)**

		**X (m)**	**Y (m)**	**X (m)**	**Y (m)**	
Set 1	Ignition	−7.48	−3.36	−7.87	−3.36	0.8
		−12.49	−3.14	−12.85	−3.13	0.6
		−17.46	−2.90	−17.78	−2.88	0.5
		−22.45	−2.67	−22.96	−2.64	0.8

	Extinction	−10.00	−3.25	−10.54	−3.21	0.8
		−14.93	−3.04	−15.63	−2.97	1.1
		−19.93	−2.78	−20.63	−2.75	1.1
		−24.93	−2.55	−25.74	−2.50	1.3

Set 2	Ignition	−8.51	−2.78	−9.22	−2.73	0.8
		−13.53	−2.51	−13.96	−2.48	0.6
		−18.47	−2.15	−18.99	−2.11	0.7
		−23.48	−1.70	−24.01	−1.65	0.7

	Extinction	−11.00	−2.64	−11.76	−2.62	0.9
		−16.01	−2.35	−16.91	−2.29	1.2
		−20.98	−1.92	−21.63	−1.87	0.9
	***Mean Delay of Ignition*** =		***0.7 s***	***Mean Delay of Extinction*** =		***1.0 s***

**Table 6.** Relative UTM positions where the perception system generated an output correction value.

**Sector 1**			**Sector 2**		

**Points (Relative UTM)**		**Correction (m)**	**Points (Relative UTM)**		**Correction (m)**
	
**X (m)**	**Y (m)**		**X (m)**	**Y (m)**	
−6.31	31.86	0.19	−38.17	−12.48	0.24
−7.67	30.47	0	−36.85	−11.22	0.28
−9.08	29.02	−0.11	−35.54	−10.08	0.30
−10.46	27.61	0	−34.29	−8.78	0.22
−11.06	27.01	0	−33.26	−7.82	−0.09
−12.43	25.68	0	−31.80	−6.48	0
−13.89	24.21	0	−30.45	−5.13	0
−15.42	22.68	0	−29.20	−3.86	0
−16.66	21.48	0.06	−27.84	−2.50	0
−18.02	20.13	−0.31	−26.33	−1.02	0.07
−19.46	18.76	0	−24.90	0.38	0
−20.74	17.43	−0.14	−23.60	1.65	0
−22.14	15.98	0	−22.24	2.98	0.11
−23.59	14.56	0	−20.81	4.44	0
−24.92	13.23	0	−19.49	5.70	0
−26.21	11.95	0	−18.12	7.03	0
−27.62	10.52	0	−16.78	8.38	0
−29.00	9.19	0	−15.54	9.65	0
−30.23	7.94	−0.12	−14.14	11.08	0
−31.68	6.49	0	−12.66	12.48	0
−32.94	5.25	0	−11.29	13.87	0
−34.34	3.81	−0.16	−9.86	15.28	0
−35.77	2.40	0	−8.51	16.68	0.06
−37.24	0.88	0	−6.98	18.18	0.09

**Table 7.** Comparison of the use of product for selective weed control.

		**Area (m**^2^**)**	**Percentage of the Coverage Area**	**Total Distance Traveled by the Four Pairs of Burners When Ignited (***Td***)**	**Total Operating Time Of the Burners (***Tt***)**	**Total Consumed Product (Kg) [35]**	**Percentage of Product Spared**
Situation 1	Total Burners treatment area	95.4	-	381.8 m	916.3 s	0.65	-
Situation 2	Low coverage weed detected	16.3	17.1%	65.3 m	156.6 s	0.17	85.1%
	High coverage weed detected	1.4	1.5%	5.8 m	13.8 s	0.02	
Situation 3	Low coverage treated area	39.1	41%	156.3 m	375 s	0.41	65.1%
	High coverage treated area	2.6	2.8%	10.5 m	25.2 s	0.04	

## Notes

**Authors Contributions** The work presented here was carried out in collaboration between all authors. Luis Emmi, Gonzalo Pajares and Pablo Gonzalez-de-Santos defined the research theme. Luis Emmi designed the architecture for the integration and synchronization of the perception, actuation, and decision making as subsystems for an agricultural autonomous system working on real wide-row crops. Gonzalo Pajares developed the algorithms for crop and weed detection. Luis Emmi and Mariano Gonzalez-de-Soto developed and implemented the algorithms for the integration and test execution. Luis Emmi analyzed and discussed the results obtained. All authors have contributed to, seen and approved the manuscript.
